# *Mycoplasma pneumoniae*-associated mild encephalitis/encephalopathy with a reversible splenial lesion: report of two pediatric cases and a comprehensive literature review

**DOI:** 10.1186/s12879-016-1985-1

**Published:** 2016-11-11

**Authors:** Norishi Ueda, Satoshi Minami, Manabu Akimoto

**Affiliations:** 1 Department of Pediatrics, Public Central Hospital of Matto Ishikawa, 3-8 Kuramitsu, Hakusan, 924-8588 Ishikawa Japan; 2 Department of Radiology, Public Central Hospital of Matto Ishikawa, Hakusan, Ishikawa Japan

**Keywords:** Encephalitis, MERS, Neuroimaging, *Mycoplasma pneumoniae*, Splenium of the corpus callosum

## Abstract

**Background:**

No literature review exists on *Mycoplasma pneumoniae*-associated mild encephalitis/encepharopathy with a reversible splenial lesion (MERS).

**Methods:**

*M.pneumoniae*-associated MERS cases were searched till August 2016 using PubMed/Google for English/other-language publications and Ichushi (http://www.jamas.or.jp/) for Japanese-language publications. Inclusion criteria were children fulfilling definition for encephalitis, *M.pneumoniae* infection, and neuroimaging showing hyperintensity in the splenium of the corpus callosum (SCC) alone (type I) or SCC/other brain areas (type II).

**Results:**

We described two children with type I and II *M.pneumoniae*-associated MERS. Thirteen cases found by the search and our 2 cases were reviewed. Mean age, male/female ratio, duration of prodromal illness was 8.3 years, 1.5 and 3.5 days. The most common neurological symptom was drowsiness, followed by abnormal speech/behavior, ataxia, seizure, delirium, confusion, tremor, hallucination, irritability, muscle weakness, and facial nerve paralysis. Fever was the most common non-neurological symptom, followed by cough, headache, gastrointestinal symptoms, headache, lethargy and dizziness. Seizure and respiratory symptoms were less common. All were diagnosed for *M.pneumoniae* by serology. Cerebrospinal fluid (CSF) *M.pneumoniae* was undetectable by PCR in the 3 patients. Three patients were clarithromycin-resistant. Leukocytosis, positive C-reactive protein, hyponatremia, CSF pleocytosis and slow wave on electroencephalography frequently occurred. All except 2 were type I MERS. Neuroimaging abnormalities disappeared within 18 days in the majority of patients. All type I patients completely recovered within 19 days. Two type II patients developed neurological sequelae, which recovered 2 and 6 months after onset.

**Conclusions:**

Prognosis of *M.pneumoniae*-associated MERS is excellent. Type II MERS may increase a risk of neurological sequelae.

## Background

Clinically mild encephalopathy/encephalitis with a reversible splenial lesion (MERS) is a clinicoradiological entity with varied etiologies, characterized by a reversible lesion with homogeneously reduced diffusion in the corpus callosum, and often associated with symmetrical white matter lesions on neuroimaging [1]. The most common causes of MERS in children are infections, including rotavirus and influenza virus [2–4]. According to the findings on neuroimaging, MERS is classified into type I involving solitary hyperintensity lesions in the splenium of the corpus callosum (SCC) and type II involving hyperintensity lesions in the SCC and other brain areas [5]. In general, the most common neurological symptom in type I MERS with varied etiologies has been reported to be delirious behavior, followed by consciousness disturbance, and seizures, all of which completely recover within a month [1].

*Mycoplasma pneumoniae* (*M.pneumoniae*) is a major cause of community-acquired pneumonia (CAP), accounting for 15–20 % of CAP cases in adults and up to 40 % of cases in children, especially in those aged 5–14 years [6]. *M.pneumoniae*-associated encephalitis is a common cause of encephalitis in children, occurring in 0.1 cases per 100,000 populations of ≤19 years of age and in 3.2–7.0 % of patients with *M.pneumoniae* infection [7–10], of which up to 64 % of cases have neurological sequelae [8, 9]. However, *M.pneumoniae*-associated MERS occasionally occurs in children, and thus clinical features including neurological symptoms in *M.pneumonia*-associated MERS remain unknown.

Here, we describe two pediatric cases of type I and II *M.pneumoniae*-associated MERS. To the best of our knowledge, the latter case of type II MERS is the first case of *M.pneumoniae*-associated MERS showing the hyperinteensity lesions in the SCC and the cerebellum. Currently, there is no comprehensive review on *M.pneumoniae*-associated MERS, and the difference in clinical features, neuroimaging findings, and outcome between type I and II MERS remains elusive. In the present study, pediatric cases of *M.pneumoniae*-associated MERS reported in the literature were searched, and a total of cases including our cases were reviewed to clarify clinical features, neuroimaging and outcome of the disease.

## Methods

### Literature search

A literature search for pediatric cases of *M.pneumoniae*-associated MERS was conducted from November 2004 till August 2016 using PubMed and Google Scholar data base for Chinese-, Croatian-, Czech-, Danish-, English-, French-, German-, Hungarian-, Italian-, Korean-, Polish-, Portuguese-, Russian-, Spanish-, and Turkish-language publications as well as using Ichushi Web data base (http://www.jamas.or.jp/) for Japanese-language publications. The search was performed using the following full keywords; ‘*Mycoplasma pneumoniae*’, ‘encephalitis’, ‘encephalopathy’, ‘magnetic resonance imaging’, ‘mild encephalitis/encephalopathy with a reversible splenial lesion’, ‘neuroimaging’ and ‘splenium of the corpus callosum’. Pediatric cases of *M.pneumoniae*-associated MERS reported in the literature and our recent cases were reviewed to clarify its clinical and demographic features, the findings on neurological imaging, and outcome. This study has been approved by the ethical committee of our institution.

### Selection criteria for case reports

Case reports were eligible and included in the analysis when they met the following inclusion criteria. Children ≤15 years of age who fulfilled; 1) the clinical definition for acute encephalitis [9], 2) the diagnosis of *M.pneumoniae* infection was confirmed by either serologic tests or PCR (polymerase chain reaction) assay for detection of *M.pneumoniae*, 3) the etiological case definition for acute encephalitis caused by *M.pneumoniae*, namely, “confirmed” (detection of *M.pneumoniae* by PCR in cerebrospinal fluid (CSF) or of intrathecal synthesis of specific antibodies), “probable” (≥4-fold rise in specific serum antibody titer using paired serum samples), or “possible” (detection of *M.pneumoniae* by PCR in throat swab specimens and/or single increased specific serum antibody titer), were considered cases [9], 4) the brain MRI revealed hyperintensity lesions in the SCC alone (type I) or in the SCC and other brain areas (type II) [5], and 5) data for demographic and clinical characteristics were reported.

### Data extraction

The following variables were extracted: patient characteristics (e.g., age, sex), acute neurological and non-neurological symptoms, duration of prodromal non-neurological symptoms prior to the onset of neurological symptoms, presence or absence of macrolide (clarithromycin) resistance defined by the absence of defervescence within 72 h after initiation of clarithromycin [11], laboratory data, including white blood cell (WBC) count in the peripheral blood, serum levels of C-reactive protein (CRP) and sodium, presence or absence of pleocytosis in CSF, findings on electroencepharography (EEG), initial and follow-up neuroimaging, duration till recovery of clinical symptoms and of abnormal findings on neuroimaging, and outcome including neurological sequelae.

## Results

### Case description

#### Case 1

A previously healthy 14-year-old boy with a 4-day history of fever and cough was referred to our hospital due to clinical deterioration despite clarithromycin treatment. He had no remarkable medical or drug history. On admission (day 1), he was alert without any neurological abnormalities. Laboratory investigations revealed normal WBC count (7,680/μL; normal range; 3,400-10,000/μL), normal blood urea nitrogen (11 mg/dL, normal range; ≤21 mg/dL), slightly elevated serum creatinine (1.03 mg/dL, normal range 0.6-1 mg/dL), hyponatremia (134 mEq/L; normal range; 135–145 mEq/L), and positive CRP (3.4 mg/dL; normal range; <0.3 mg/dL). Serum levels of calcium, magnesium, glucose and the liver function test were normal. Serum anti-*M.pneumoniae* IgM antibody using a rapid enzyme immunoassay (EIA, Immunocard® Mycoplasma, Meridian Bioscience Inc., OH, USA) was negative. Antigens of influenza virus and adenovirus in the throat swab specimens were negative. Urinalysis was normal. Analysis of CSF was not performed.

**Fig. 1:**
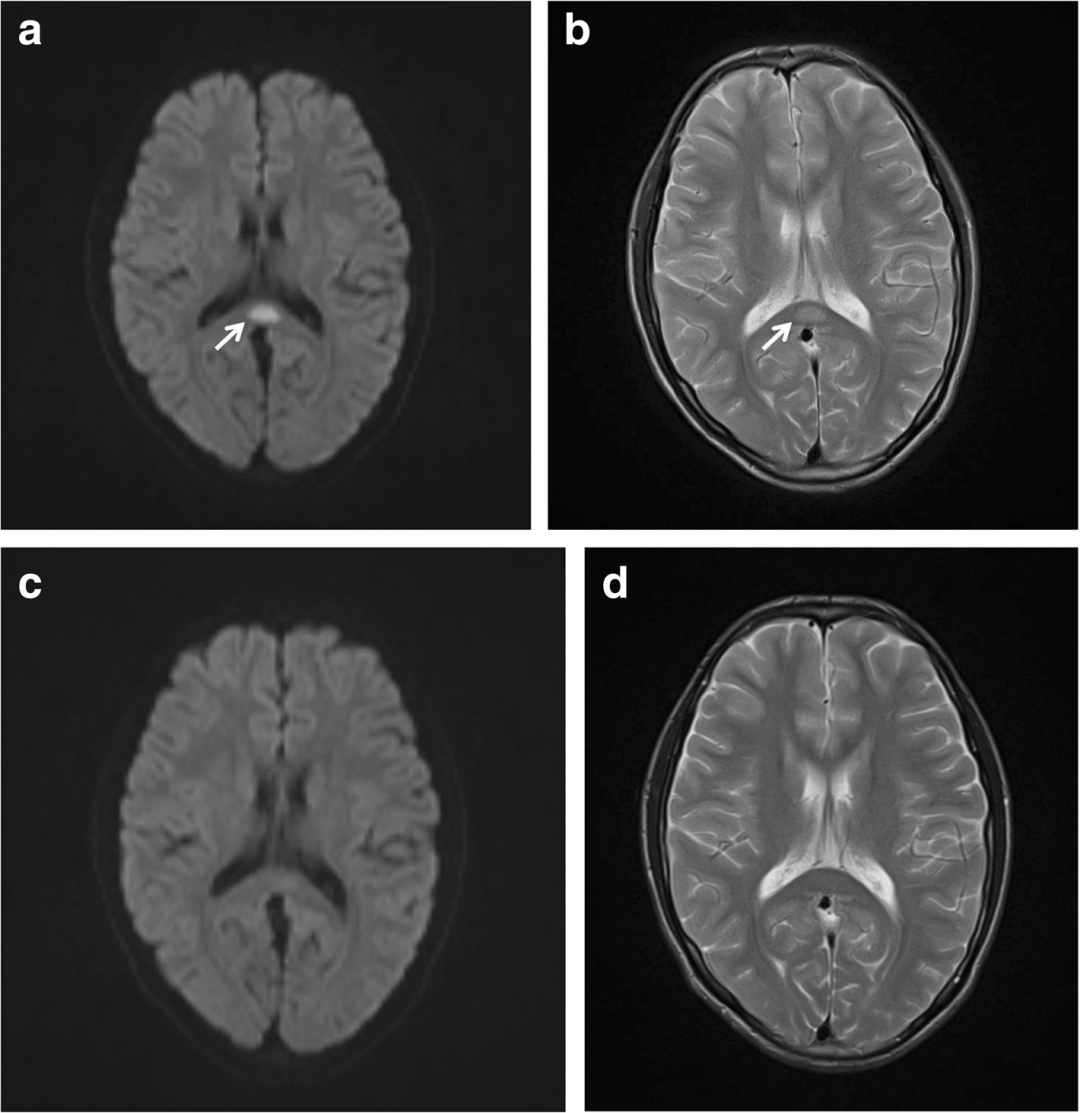
The brain magnetic resonance imaging (MRI) in case 1. The brain MRI on day 3 after admission revealed high intensity lesions (arrows) in the splenium of the collupus callosum (SCC) on diffusion- (**a**) and T2- weighted images (**b**), which disappeared on day 7 (**c** and **d**)

A chest X-ray revealed dense infiltration in the bilateral lower lobes, indicative of pneumonia. Intravenous minocycline (100 mg/day) was administered for the treatment of *M.pneumoniae* pneumonia. In the following evening, he became afebrile but developed abnormal speech and hallucinations. In the morning on day 3, he suddenly developed delirious behavior followed by drowsiness. Glasgow Coma Scale (GCS) score was 8 (E3, V1, M4). The brain MRI revealed hyperintensity lesions in the SCC on diffusion- and T2-weighted images (Fig. 1a and b). Intravenous dexamethasone and acyclovir were administered. He rapidly improved and was fully conscious in the evening on day 3. Neuroimaging on day 7 revealed disappearance of hyperintensity lesions in the SCC (Fig. 1c and d). Laboratory investigations revealed negative CRP, while seroconversion of serum anti-*M.pneumoniae* IgM antibody was noted. He was discharged without neurological sequelae on day 7.

#### Case 2

A previously healthy 8-year-old girl with 1-day history of cough, headache, fever, lethargy, vomiting and diarrhea followed by drowsiness and seizures for ~20 s was referred to our hospital. She had no remarkable medical or drug history. On the same day, her younger brother was admitted to our hospital because of pneumonia due to *M.pneumoniae* diagnosed by pulmonary manifestation, chest X-ray finding, and positive *M.pneumoniae*-specific IgM. On admission (day 1), she was drowsy; GCS score was 8 (E3, V1, M5). Her body temperature was 38.3 °C, and the blood pressure was normal (108/68 mmHg). Physical examination revealed neither nuchal rigidity nor neurological abnormalities. Laboratory investigations revealed that WBC count (6,480/μL), serum levels of CRP, calcium, magnesium, glucose, the liver function test and urinalysis were within the normal range. However, hyponatremia (132 mEq/L) and positive serum anti-*M.pneumoniae* IgM antibody were noted. Serum IgM antibody against Epstein-Barr virus capsid antigen was negative but IgG antibody was positive, indicative of previous infection. Antigens of influenza virus and adenovirus in the throat swab specimens, and those of rotavirus, norovirus and adenovirus in the stool specimens were negative. Stool culture revealed no pathogenic bacteria. Analysis of CSF was not performed.

**Fig. 2:**
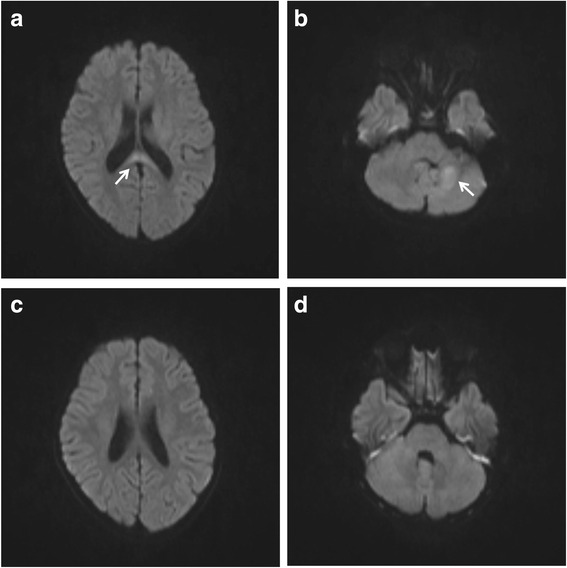
The brain MRI in case 2. The brain MRI on day 5 revealed high intensity lesions (arrows) in the SCC (**a**) and the left cerebellum (**b**) on diffusion-weighted images, which disappeared on day 12 (**c** and **d**)

A chest X-ray revealed increased bronchial markings in the right lower lobe. The brain computed tomography (CT) and the EEG revealed no abnormalities. Intravenous fluid therapy was immediately given, followed by administration of an anticonvulsant, diazepam suppository (6 mg), for prophylaxis of further convulsions. Six hours later, her consciousness was fully recovered. On the following day, she started receiving oral minocycline (2 mg/kg/day). On day 5, she suddenly developed headache, drowsiness, ataxia and intension tremor. The brain MRI revealed hyperintensity lesions in the SCC (Fig. 2a) and the left cerebellum (Fig. 2b) on diffusion-weighted images. Similar signal characteristics were noted in the SCC and the left cerebellum on T2-weighted images.

Oral minocycline was switched to intravenous administration for additional 3 days. On day 6, she became afebrile, rapidly improved, and was fully conscious without disturbance of gait, cognition, speech, swallowing and vision. However, she still had intension tremor, slight muscle weakness and disabled fine motor incoordination of the left hand. Neuroimaging on day 12 revealed disappearance of the hyperintensity lesions in the SCC and the left cerebellum (Fig. 2c and d). She was discharged on day 14 with slight disability of fine motor incoordination of the left hand, which completely recovered 2 months after the onset of the disease.

### Litterature review

The literature search found 13 cases, including 4 English-language full reports (7 cases) [5, 12–14], 1 Japanese-language full report (1 case) [15] and 5 Japanese-language abstract-only reports (5 cases) [16–20]. Our recent 2 cases described above were added for a total of 15 cases with *M.pneumoniae*-associated MERS. Of the 15 patients, 5 and 10 patients were “probable” and “possible” cases, respectively, while none was “confirmed” case. Demographic, clinical characteristics, laboratory data, neuroimaging findings, and outcome of these patients are summarized in Table 1. Mean age, male/female ratio, and mean duration of prodromal illness before the onset of neurological symptoms were 8.3 years (range 2–14 years), 1.5 (9/6), and 3.5 days (range 1–8 days), respectively. Of note, mean period of prodromal illness before the onset of neurological symptoms was shorter in the patients with type II MERS (1 day) than in those with type I MERS (3.8 days, range 1–8 days).

**Table 1:** Pediatric cases of MERS associated with *Mycoplasma pneumoniae* infection

Age (yrs)	Sex	Duration of prodromal illness (d)	Neurological symptoms	Non-neurological symptoms	Duration of fever (d)	Diagnosic method for MP	Diagnosis of MP-associated encephalitis	MR	Chest	WBC	CRP	Na	CSF	EEG	MRI	Time till recovery of MRI findings (d)	Time till clinical recovery (d)	Treatment		Outcome	Ref
									X-ray/CT	(cells/μL)	(mg/dl)	(mEq/L)	finding		findings			Antibiotics	Steroids		
13	M	1	Abnormal speech, ataxia, confusion, drowsiness	Abd pain, fever, lethargy	NA	IgMAb/CFT	probable	NA	NA	7,950	0.1	NA	pleocytosis	SW	type II, SCC, center semiovale, genu, parietal	90	180	CIP	–	CR	[5]
3	F	3	Drowsiness, seizure	Diarrhea, fever, vomiting	6	IgMAb/EIA	possible	NA	NA	normal	–	NA	normal	NA	type I	60	3	AZT	–	CR	[12]
8	M	2	Ataxia, confusion, drowsiness	Fever, lethargy	6	IgMAb/EIA	possible	NA	NA	increased	+	NA	normal	NA	type I	6	8	AZT	+	CR	[12]
9	M	1	Drowsiness, left peripheral facial nerve paralysis	Fever, headache, lethargy, vomiting	2	IgMAb/ELISA MP (+) in PS by PCR	possible	NA	NA	4,500	1.6	127	normal MP (−) in CSF by PCR	SW	type I	7	10	AZT	–	CR	[13]
12	M	6	Drowsiness	Cough, dizziness, fever, headache, lethargy, vomiting	6	IgMAb/ELISA MP (+) in PS by PCR	possible	NA	IF	8,100	1.4	139	normal MP (−) in CSF by PCR IL-6↑	SW	type I	3	8	AZT	–	CR	[13]
2	M	2	Abnormal speech, irritability, motor deterioration, seizure	Abd.pain, fever, vomiting	2	ELISA	possible	NA	NA	11,600	6.6	136	normal	normal	type I	120	8	NA	–	CR	[14]
6	M	4	Abnormal speech, delilium, drowsiness	Abd.pain, fever, headache	4	ELISA	possible	NA	NA	12,600	16.8	135	normal	SW	type I	67	19	NA	+	CR	[14]
6	M	6	Drowsiness, irritability	Cough, fever, headache	10	PA	probable	+	IBM	11,800	0.33	135	pleocytosis	SW	type I	18	12	CIP	–	CR	[15]
8	F	1	Ataxia, muscle weakness, tremor	Cough, fever	NA	PA	probable	NA	IF	8,000	5.72	136	ND	NA	type I	8	8	NA	NA	CR	[16]
10	F	1	Drowsiness	Cough	0	PA	probable	NA	IF	NA	NA	NA	ND	NA	type I	15	4	NA	NA	CR	[17]
12	F	7	Abnormal behavior, drowsiness, seizure	Fever	NA	NA	possible	NA	NA	NA	NA	NA	NA	NA	type I	NA	10	NA	+	CR	[18]
6	F	4	Delirium	Cough, fever	8	NA	possible	+	IF	NA	NA	NA	ND	NA	type I	11	8	CIP	NA	CR	[19]
7	M	8	Abnormal speech, drowsines	Cough, fever, headache	6	PA	probable	NA	NA	16,400	1.1	131	pleocytosis MP (−) in CSF by PCR	normal	type I	4	4	MINO	+	CR	[20]
14	M	5	Abnormal speech/behavior, delirium, drowsiness, hallucinations	Cough, fever, headache	5	IgMAb/EIA	possible	+	IF	7,680	2.6	134	ND	ND	type I	7	7	MINO	+	CR	Case1
8	F	1	Ataxia, drowsiness, intension tremor, seizure	Cough, diarrhea, fever, headache, lethargy, vomiting	9	IgMAb/EIA	possible	NA	IBM	6,480	<0.3	133	ND	normal	type II,SCC, left cerebellum	7	60	MINO	–	CR	Case2

*Ab* antibody, *Abd* abdominal, *AZT* azithromycin, *CFT* complement fixation test, *CIP* ciprofloxacin, *CR* complete recovery, *CRP* C-reactive protein. *CSF* cerebrospinal fluid, *CT* computed tomography, *EEG* electroencephalography, *EIA* enzyme immunoassay, *ELISA* enzyme-linked immusorbent assay, *IBM* increased bronchial markings, *IF* infiltration, *IL* interleukin, *MINO* minocycline, *MERS* mild encephalitis/encephalopathy with a reversible splenial lesion, *MP Mycoplasma pneumoniae*, *MR* macrolide (clarithromycin) resistance, *MRI* magnetic resonance imaging, *PA* particle agglutination assay, *PCR* polymerase chain reaction, *PS* pharyngeal swab sample, *SCC* splenium of the corpus callosum, *SW* slow wave, *WBC* white blood cell. ↑ increase, *ND* not done, *NA* not available

As neurological symptoms, drowsiness (12/15, 80 %) was the most common, followed by abnormal speech (5/15, 33 %), ataxia (4/15,27 %), seizure (4/15,27 %), delirium (3/15, 20 %), abnormal behavior (2/15,13 %), confusion (2/15,13 %), tremor (2/15, 13 %), irritability (2/15,13 %), hallucination (1/15,7 %), muscle weakness (1/15, 7 %), motor deterioration (1/15, 7 %), and peripheral facial nerve paralysis (1/15, 7 %). Among non-neurological symptoms, fever (14/15, 93 %) was the most common, followed by cough (8/15, 53 %), headache (7/15, 47 %), gastrointestinal symptoms (abdominal pain, vomiting and diarrhea, 6/15, 40 %), lethargy (5/15, 33 %), and dizziness (1/15, 7 %). Prolonged fever (≥6 days) developed in 58 % (7/12) of the patients, in whom the information of duration of fever was available. Seven (47 %) of the 15 patients had no respiratory symptoms.

All patients were diagnosed by serologic tests. Eight (53 %) of the 15 patients were diagnosed by a single measurement of *M.pneumoniae*-specific antibody; there were 4 and 2 cases with positive IgM antibody as measured by EIA and enzyme-linked immunosorbent assay (ELISA), respectively, and in the remaining 2 patients, *M.pneumoniae*-specific antibody as measured by ELISA was positive but what type of antibody was unknown. Of these 8 patients with positive *M.pneumoniae*-specific antibody, 2 patients simultaneously showed positive *M.pneumoniae* DNA in the throat swab specimens by PCR. There was a 4-fold or more rise in serum anti-*M.pneumoniae* antibody titer as measured by complement fixation test (CFT) in 1 case and by particle agglutination assay (PA) in 4 cases. Information of the method for serologic tests was not available in the remaining 2 cases. None of the patients underwent culture analysis.

Three patients showed clarithromycin resistance. Chest X-ray or CT of the 7 patients examined revealed infiltration of the lobes (*n* = 5) or increased bronchial markings (*n* = 2). Leukocytosis (>10,000 WBCs/μL), positive CRP (>0.3 mg/dL) and hyponatremia (<135 mEq/L) were noted in 42 % (5/12), 75 % (9/12) and 44 % (4/9) of the patients, respectively. CSF pleocytosis (>10WBCs/μL) was found in 3 (33 %) of the 9 patients, three of whom showed negative *M.pneumoniae* in CSF as measured by PCR. The levels of interleukin (IL)-6 in CSF were elevated in a patient with type I MERS. EEG revealed slow wave in 63 % (5/8) of the patients examined.

On neuroimaging, all except 2 patients showed type I MERS. Two patients were type II MERS; our case 2 with hyperintensity lesions in the SCC and the left cerebellar lesions, and other case with those in the SCC, center semiovale and parietal white matter bilaterally [5]. Hyperintensity lesions in the SCC and other brain areas on neuroimaging disappeared within 18 days in all except 4 patients including 3 with type I and 1 with type II MERS, in whom the lesions on the follow-up MRI disappeared 2–4 months after the initial MRI.

Antibiotic treatment included azithromycin (4/10), ciprofloxacin (3/10), and minocycline (3/10), while none received clarithromycin after the onset of MERS. Intravenous steroids were given to 42 % (5/12) of the patients. All patients with type I MERS completely recovered within 19 days, while 2 patients with type II MERS developed neurological sequelae, which recovered 2 and 6 months, respectively, after the onset of the disease.

## Discussion

There is some concern about the diagnostic tests for acute *M.pneumoniae* infection. Culture is impractical since the long time is required to get the results [21]. Serologic tests have been most widely used and a 4-fold rise in antibody titer in acute and convalescent sera is considered the “gold standard”. However, the use of a single qualitative measurements of IgM has low sensitivity (32–77 %)[22, 23], which increases (88.6 %) when paired sera are analysed [23]. Taking sera during both phases is too late for point-of-care diagnosis and difficult in children [22]. False-positive results occur since IgM remains detectable for several months following infection [21, 22]. False-negative results occur when serum is collected within 7 days after onset [21, 22] and in immunocompromised patients　and infants <6 months of age, who often do not produce IgM [21]. PCR is highly sensitive and measures blood, CSF, and pharyngeal samples. False positive results occur due to colonization and prolonged shedding from previous infection [22]. False-negative results occur due to contamination, inhibitors in samples, or timing of sample collection [22]. Thus, the combination of PCR plus serology yields the most reliable results. Despite inadequate validity of serology and untested other respiratory pathogens, pulmonary symptoms, chest X-ray findings, IgM seroconversion and interfamilial *M.pneumoniae* infection episode strongly suggest that our cases are considered “possible” cases.

All except one case [5] were reported from Asia; Japan [12, 15–20] and China [13, 14], suggesting a role of racial factor(s) for *M.pneumoniae*-associated MERS. As *M.pneumoniae*-associated encephalitis [7–10], *M.pneumoniae*-associated MERS predominantly occurs in children aged 2–14 years. Adult case of *M.pneumoniae*-associated MERS was only reported [24]. Thus, young age may be a predisposing factor of *M.pneumoniae*-associated MERS. As *M.pneumoniae*-associated encephalitis [9] and MERS by other causes [14], male preponderance is noted in *M.pneumoniae*-associated MERS.

Extrapulmonary complications, in particular encephalitis, occurred more frequently in children with macrolide-resistant than in those with macrolide-sensitive *M.pneumoniae* [25]. However, our study suggested that clarithromycin resistance is not a predisposing factor of *M.pneumoniae*-associated MERS.

Disturbance of consciousness, delirious behavior and ataxia are common neurological symptoms in *M.pneumoniae*-associated MERS as MERS by other causes [3, 26]. In contrast to *M.pneumoniae*-associated encephalitis (48–67 %) [8, 27, 28], seizure is less common (27 %) in MERS due to *M.pneumoniae* as that by other causes (14–32 %) [3, 26], probably due to mild brain dysfunction in *M.pneumoniae*-associated MERS. This is supported by the EEG finding; normal to mild abnormality (i.e. slow wave) as in MERS by other causes [26] versus diffuse cortical dysfunction and focal epileptiform discharge in *M.pneumoniae*-associated encephalitis [27, 29]. It remains unknown how the SCC lesions cause neurological manifestations. The splenium is the posterior part of the corpus callosum, connecting different cortical areas, including occipital, parietal and temporal lobes [30]. Thus, the lesions in the SCC and other brain regions connecting SCC lead to various neurological manifestations [31]. Neuroimaging findings in type II MERS suggests that neurological symptoms in MERS may be due to the lesions in both the SCC and other brain regions connecting the SCC.

Fever is the most common non-neurological symptom as in *M.pneumoniae*-associated encephalitis [7–10, 29]. Prolonged fever (≥6 days) is noted in the majority of the patients, while respiratory symptoms are less common (47 %) as in *M.pneumoniae*-associated encephalitis (~44 %) [7, 32]. Longer intervals between respiratory and CNS manifestations were associated with worse outcome in *M.pneumoniae*-associated encephalopathy [33]. However, short intervals in type II *M.pneumoniae*-associated MERS suggest that short period of prodromal illness before the onset of CNS manifestations may predict severe MERS as in *M.pneumoniae*-associated encephalitis [8].

Leukocytosis and positive CRP were associated with severe *M.pneumoniae* infection [34]. Prevalence of leukocytosis and positive CRP in the patients reported here is similar to that (57 and 71 %, respectively) in MERS by other causes [3]. Our and other cases of type II MERS [5] showed no leukocytosis and negative CRP, suggesting no predictive values of these parameters for worse outcome. Our study showed that CSF pleocytosis occurs more frequently in *M.pneumoniae*-associated MERS (33 %) than in MERS by other causes (0 %) [3], and its prevalence is similar to that (33–66 %) in *M.pneuminiae*-associated encephalitis [8, 29, 35]. In contrast to *M.pneumoniae*-associated encephalitis [35], CSF pleocytosis does not seem to increase a risk of neurological sequelae or worse outcome.

The mechanism by which the SCC lesions occur in MERS remains elusive. Hyponatremia has been proposed to reduce the intracellular osmotic pressure in the SCC, leading to transient edema [36]. Hyponatremia exacerbates the SCC injury [37]. However, hyponatremia occurs in less than a half of *M.pneumoniae*-associated MERS patients, similar to MERS by other causes (37–83 %) [3, 14, 26, 36, 38], suggesting that hyponatremia may be a modulator but not essential for development of the SCC lesions. As *M.pneumoniae*-associated encephalitis [7, 9, 33], PCR rarely detected *M.pneuminiae* in CSF of *M.pneumoniae*-associated MERS [13, 20], suggesting indirect mechanisms such as immune-mediated mechanism [1] rather than direct invasion of *M.pneumoniae,* leading to the SCC lesions. The levels of CSF IL-6 were increased in MERS due to *M.pneumoniae* [13] and other causes [39]. IL-6 induces the SCC injury [40]. An oxidative stress marker in CSF was increased in MERS by other causes [39]. Low levels of glutathione reductase, anti-oxidant enzyme, in the corpus callosum [41] make the SCC more susceptible to oxidant injury. Proinflammatory cytokines, oxidants and reduced anti-oxidant enzymes may lead to the SCC lesions. Anti-N-methyl-D-aspartate receptor (NMDAR) antibody was detected in serum and CSF of *M.Pneumoniae*-associated encephalitis [42]. NMDARs are expressed in the SCC [43]. Thus, anti-NMDAR antibody may be a potential contributing factor to the SCC lesions [44].

*M.pneumoniae*-associated MERS is type I in almost all patients, while 2 (13 %) patients developed type II MERS [5] including our case. In type I MERS by other causes, hyperintensity lesions in the SCC disappeared 3 days to 2 months following the initial MRI and 53 % of the patients recovered within 1 week [45]. In *M.pneumoniae*-associated MERS, the neuroimaging finding normalized ~18 days after the initial MRI in all except 4 (3 type I and 1 type II) [5, 12, 14], in which it normalized 2–4 months.

As MERS by other causes (31 %) [25], intravenous steroids were only given to 42 % of the patients. Steroids are not essential other than treating concomitant infection partly because the natural history of *M.pneumoniae*-associated MERS is almost always excellent.

Despite high mortality (9 %) [10] and prevalence of neurologic sequelae (18–64 %) in *M.pneumoniae*-associated encephalitis [8, 10, 28], prognosis is excellent and neurological sequelae never develop in type I *M.pneumoniae*-associated MERS. All except 2 patients with type II MERS [5], including our case, fully recovered within 19 days after the onset. Neurological symptoms recovered as the neuroimaging finding improved. In contrast, our case and other case with type II MERS [5] developed neurological sequelae, which disappeared 2 and 6 months, respectively, after the onset, suggesting that prognosis of *M.pneumoniae*-associated MERS may depend on the extent of brain lesions affected.

Limitation of the present study includes its retrospective and observational nature, too small sample size, and inadequate validity of diagnostic method for *M.pneumoniae* infection in reported cases.

## Conclusions

MERS could be associated with *M.pneumoniae* infection and *M.pneumoniae*-associated MERS predominantly occurs in children with male preponderance. Seizure and respiratory symptoms are less common. Despite excellent outcome in type I *M.pneumoniae*-associated MERS, there may be a risk of neurological sequelae in type II MERS, depending on the brain lesions affected. Short intervals between prodromal illness and CNS manifestations may be associated with type II MERS. Limitation of the present descriptive and retrospective study with small sample size warrants further investigations to clarify clinical features and risk factors of MERS that could be associated with *M.pneumoniae* infection.

## Abbreviations

CAP: Community-acquired pneumonia
CFT: Complement fixation test
CNS: Central nervous system
CRP: C-reactive protein
CSF: Cerebrospinal fluid
CT: Computed tomography
EEG: Electroencephalography
EIA: Enzyme immunoassay
ELISA: Enzyme-linked immunosorbent assay
GCS: Glasgow coma scale
IL: Interleukin
*M.pneumoniae*: *Mycoplasma pneumoniae*
MERS: Mild encephalitis/encephalopathy with a reversible splenial lesion
MRI: Magnetic resonance imaging
NMDARs: N-methyl-D-aspartate receptors
PCR: Polymerase chain reaction
SCC: Splenium of the corpus callosum
WBC: White blood cell
